# Book Reviews

**Published:** 1807-08

**Authors:** 


### CRITICAL ANALYSIS
### OF THE
### RECENT PUBLICATIONS
### ON THE
### DIFFERENT BRANCHES OF PHYSIC, SURGERY, AND MEDICAL PHILOSOPHY.

---

### *The Edinburgh Medical and Surgical Journal, No. XI.*

Article 1.—*Biographical Sketch of Camper.*

As this celebrated professor has now been near 20 years dead, the rising generation may feel less interest in the few memoirs his life affords. Like many other philosophers, little more can be said of him, than what his works proclaim. " He seems, says the author of this Sketch, to have followed the common route among men of letters, and left few memorials of his happiness or misfortunes." This account might form a very proper introduction to a collection of his physiological works, and from such it has been extracted.*

Article 2.—*History of a Case of Introsusception, with Remarks on the Complaint.* By George Langstaff, Surgeon, London.

This paper does the author no inconsiderable credit; but we wish it had been more free from what is very expressively called *showeyness.* It is true, in the description of *introsusception,* in its divisions into the inner descending, the middle reflecting, and the external containing fold, and also the two angles of reflection: we have in the latter paragraph, a reference to a *representation* given by Mr. Hunter in the Chirurgical Transactions ; but unless the reader turns to the passage, he will not be aware that the whole account is almost transcribed from that paper.

However, we must admire the modesty of the author, who conceives it " presumptuous in him, to comment on any of the remarks of that luminary, Mr. Hunter." If Mr. Hunter is to be considered as such a luminary, we conceive he must have had more sense than to be flattered by silent assent without comment, and would gladly have received a hint, especially in diseases, which, as our author observes, " belong more to the department of an apothecary than a surgeon." But if the disease, when not relieved by the efforts of Nature, admits of no remedy, it is of little consequence to distinguish it from inflammation, which Dr. Baillie has not attempted † to do. All that we can undertake, is to

---

* Œuvre de Pierre Camper, Paris, 1803.

† Morbid Anatomy, 3d Edit, Page 200.

to abate inflammation, the better to enable Nature to accomplish the developement of the parts.

However, if a writer whom we thought worthy to be called a luminary, had proposed any other attempts, we should have hesitated before we expressed our regret, that "so universally and justly respected a name should be affixed to a proposal so manifestly absurd."

Article 3.—*Case of a Man who died in consequence of a Disease occasioned by what may in general be considered as a Mild Punishment.* By M. BURMESTER, Army Surgeon.

THERE is nothing very remarkable in this case, inasmuch as death by mortification, has often followed injuries much less considerable than the infliction of *two hundred and fifty* military lashes. The paper is on other accounts valuable, and the motives of the author extremely praise worthy. It would have added something to the interest we felt in the perusal, had an account been given of the temper and disposition of the subject. We are persuaded that the disgrace attending such a punishment, would in many be a sufficient means of accounting for so fatal an issue.

Article 4.—*Account of the Ockow, a Chinese Remedy for Complaints in the Breast, with Observations on the Use of Digitalis.* By A. BAILDON, M. D. London.

THE author supposes the first remedy to be animal gluten. One remark on digitalis, from the effects he found in his own person, will we hope be attended to by those practitioners who are attached to this powerful remedy.

"I suspect, says he, that the difference in the accounts we have of it by different practitioners, has arisen from a circumstance very clearly marked in my case, and which strongly points out the great care and attention necessary in exhibiting this very active drug. I observed, and repeated the experiment a great many times, that after the digitalis had taken effect, *my pulse was not lessened in frequency when I stood erect;* it was then upwards of 100. When I sat down, it fell considerably; when lying on my back, it fell much more. Thus, during the time it was at 40 when lying, it was about 75 when sitting, and above 100 when standing. This was invariably the case. When I turned on either side, it fell two or three, and intermitted.

"I have found the pulse to vary in this manner, in all the patients to whom I have given the digitalis to any extent. I need hardly point out the absolute necessity of carefully attending to this circumstance."

Article 5.—*On the Tic Douloureux, or Trismus Dolorificus.*

THIS article gives an account of two cases, in which Dr. Pierson succeeded in curing this very painful disease by mercurial salivation; a third, in which the patient died of the remedy; and a fourth, in which the patient declined its use.

Article 6.—

Article 6.—*Report on the Malignant Disease which prevailed in the City of New-York, in the Autumn of* 1805, *addressed to the Governor of the State of New-York.* By EDWARD MILLER, Resident Physician in the State of New-York.

THIS article, consisting of twenty-one pages, has already appeared in our Journal, No. 96, Vol. xvii. Page 97.

Article 7.—*Cases of Small-pox in the Fœtus.* By WILLIAM FORBES, Member of the Royal College of Surgeons, London.

THIS paper contains three cases very worthy of being recorded.—The first is of a woman, who at the sixth month of her pregnancy passed through confluent small-pox, and was afterwards delivered at the natural period. It appears by the future history of the child, that it was not infected *in utero*, which may account for the mother going her full time.

The second is, we believe, the first instance on record, of a child born with small-pox, and surviving. We wish the case had been related with more minuteness, as to the periods of the disease in the mother and child, connected with the birth of the latter.

In the third case, the mother had passed through the small-pox in her infancy. During pregnancy, she nursed a son with small-pox, who died. The child born, had small-pox pustules at the time of its birth. This was in the year 1788, and at this time, being in her nineteenth year, she has on her face and other parts of the body incontestible proofs of the disease. By this, it appears probable, as has been remarked by Dr. Adams, that the appearance of pock-marks in the face only, is to be ascribed to the greater inflammation attending pustules in that part, from its receiving the first shock of the disease, consequently that in the fœtus every part being similarly circumstanced, will receive the impression at the same time, and in the same manner.

In both these cases, if the author has any further memoranda relative to the dates of the different events, or the degree in which the children were affected, they will add much to the value of his communication.

Article 8.—*Report of the General Hospital, near Nottingham, from March* 25, 1806, *to March* 25, 1807. By JAMES CLARKE, M. D. one of the Physicians to the Charity.

THE original articles finish with a short conclusion to the Enquirer's paper on the Royal Touch.

We are truly sorry to be obliged to remark the striking difference in the *Critical Analysis*. It requires no ingenuity to see that those venerable names who did so much credit to the *Annals*, have relinquished that important office. During *their* labours, we met with no criticisms, without stating the author's meaning; no unprovoked pertness where the intentions of their author were good; no surreptitious invasions of their author's matter delivered as their own. We would advise these truly respectable editors, no longer to shelter themselves under the veil of an anonymous publication.

Let them reflect on the dignity of their own character, and if the labour of reviewing is too considerable, at least inspect the productions of those whom they employ. Let them reflect on the disgrace which a contemporary Journal of the same meridian incurred by the partial manner in which medical articles were reviewed. Fond as the public may seem of a severity to which they are in no fear of being exposed, there are limits which may be exceeded. The editor of the Edinburgh Review seems conscious that he can no longer trust his writers with medical works; and it is notorious, that the only article of that description, which has for some time appeared, was written by himself, though not of the profession. We forbear to dwell on so unpleasant a subject; but the respect we bear the profession, and the high opinion we entertain of the well known editors of the work before us, oblige to say thus much, and we sincerely hope it will not be necessary to say more.

---

*Dissertations on Man, Philosophical, Physiological, and Political. By* J. Jarrold, M. D. 8vo.

This work being intended as a subject of political œconomy, it may be thought, might have passed unnoticed in our Journal. But the physiological part contains some remarks entirely new, and not less ingenious. We feel ourselves, therefore, impelled to bring them before the faculty, that they may meet with that general attention from individual observations, by which only they can be confirmed or refuted.

Few of our readers can be ignorant of the valuable Treatise on Population presented to the public by the Rev. Mr. Malthus. One very important part of that gentleman's doctrine seems to explain the laws by which the population of mankind is kept under by vice and misery, and even to show that such means are not unnecessary to prevent the race from eating up one another. Dr. Jarrold's view of the subject is infinitely more consolatory, and, in our opinion, not less satisfactory to common sense than honourable to that Fountain of good, from whom only natural laws can originate. After considering the various causes which prevent population in those different countries in which Mr. Malthus has traced them, and pointing out various errors of that gentleman, our author enters on the subject as a physiological question.

He first shows, that the longevity of the Antediluvians was absolutely necessary, in order that arts of any kind useful to man, might be brought to such a state as to facilitate their future improvement; confirms the tradition, that after the flood human life was gradually shortened till it arrived at the period marked out by David, which he conceives may still be considered as the proper medium, where no particular events tend to shorten its duration. He then enters on the checks which are opposed to a redundant population, and shows shows that they are no way inconsistent with the virtue, happiness, and good order of society.

Women, he remarks, as well as other animals, are only capable of fecundation at certain periods. These periods are more various in all animals in proportion as they become more domesticated, and probably in women, as their mode of life becomes more civilized or artificial. Should any occurrence, bodily or mental, happen at such a period, the probability is shown that it may supercede an event which would otherwise take place by interrnpting those gradual changes in the constitutiou which are necessary, in order to render it prolific. Various other causes are shown to be more or less productive of sterility; but most of all, it would seem, that whether the mind is occupied in its own distresses, or deeply engaged in any other pursuits, whether of science or ambition, the effect on the mere animal organs is the same.

"In civilized society," says our author, "instances are common of a family that promised to be numerous being stopped in its progress by some circumstance of distress preying on the minds of the parents. It seldom happens that an honest man is a parent in the year in which he becomes a bankrupt.

"There is, in this and other countries, a certain class of unhappy persons, in whom virtue is not blended with misfortune, or a stained character the effect of unavoidable circumstances, which still more unequivocally shows the influence of the mind in preventing pregnancy, I mean, that of the prostitute. It is well known that a prostitute is seldom a mother, which is usually attributed to promiscuous intercourse, but this is not the cause: to suppose that a young woman at once leaves the paths of virtue and the protection of her parents for infamy, disease and want, is as unreasonable as it is unjust; language, offensive to the ear of modesty, is first heard and endured, and afterwards the life by degrees becomes corrupt. At the very outset of a course of vice, whilst perhaps only an individual is a party in the crime, barrenness is almost as common as in the more openly profligate. To state a case; a woman lives with her paramour, the ceremony only of a public acknowledgment is not complied with to constitute them man and wife: No, they stand in no such relation; the laws of the country are not observed, for the express purpose that this relationship may never be felt. Theirs is an union of criminal desires, not of honourable motives: the crime constitutes their happiness. Were a law passed, by which such conduct was made to constitute marriage, the principle of their union would be dissolved: the source of such happiness is personal gratification, the source of happiness in marriage is personal worth. The interests of a mistress are centered in herself; abandoned by her own sex, and degraded by the other, the objects of her attention are as limited as those of a savage; and like a savage she spends her days, in fanning a passion, which, the more it reigns,

 the the more despicable she becomes, and the more disqualified for being a mother.

"That the class of Women of which I have been speaking owe their sterility to the influence of their minds, is considerably strengthened by the consideration, that promiscuous intercourse is not of itself a hinderance to pregnancy. At Otaheite, and many other places, where the manners of the people admit of this practice without incurring disgrace, no check to population follows: they have no proper sense of shame; their conduct does not appear to them corrupt; their intercourse is precisely that of brutes, and brutes are never sterile because of promiscuous intercourse; it has no injurious effect in the propagation of animals.

"In Europe the female character is held in the highest estimation, and is as sacred as life itself; but at Otaheite, or among savages, if virtue has a name, the sentiment is wholly unknown: a sense of decency characterizes civilization. In Europe the female takes alarm at the most distant insinuation, and days of the severest conflict precede and accompany a dissolute life; the mind is never at rest: but the unrestrained manners of an unenlightened people are never checked by reflection. Unrestrained manners in an European are not the mere indulgence of animal instinct, they imply the triumph of depravity over virtue and religion; the struggle is severe: it is the mind that is conquered; it had modelled the face to be an index of itself, the index remains, but the character it expresses is changed: the new aspect given by depravity demonstrates the influence the mind has over the body. If the mind affects the external character, it doubtless biasses the constitution."

Our author next considers the state of the mind as influencing the constitution, without any particular view to moral depravity, or the common causes of abject distress, and by a happy quotation from the illustrious Adam Smith, shows, that the difficulties by which a family is continued, multiply in proportion as a state advances to civilization.

"It is," says Dr. J. "a familiar fact, that the higher class is unproductive: but the important part of the fact is, that this circumstance is limited to civilized nations; it extends no further than where superiority in rank implies superior information. The chiefs of a nation of shepherds are not unprolific; in all nations in that state, or even advanced a step further, genealogies reaching down many centuries are common. "I cannot help remarking it," says Dr. Smith, "that very old families, such as have possessed some considerable estate from father to son for many successive generations, are very rare in commercial countries. In countries which have little commerce, on the contrary, such as Wales, or the Highlands of Scotland, they are very common. The Arabian historians seem to be all full of genealogies: and there is a history, written by a Tartar, Kham, which has been translated into several European languages, and which contains scarcely any any thing else: a proof that ancient families are very common among those nations." *Wealth of Nations*, *v.* 2, *p.* 129.

"Such authority, for the general fact, may be considered as conclusive, but the reasoning admits of doubt: the change of property in a commercial country is certainly frequent; but it is not wholly in consequence of the rise and fall of fortunes which commerce gives birth to. Athens was not a commercial city, nor Berne, and yet old families were rare. The nobility of this kingdom are above the influence of commerce, yet they cannot boast of estates being many centuries in their families. It is not from commerce only that such events originate; they must have another cause. A commercial country is always civilized; commerce is in part the cause and the consequence of civilization, but the uncertainty that attends it is not the sole occasion of the frequent changes of property. Estates made hereditary often want an heir, and this I apprehend to be the principal reason that families hold large estates for so short a time. Those who enjoy any rank in a civilized country, who stand at its head, either in intellect or in power, stretch their capacity to the utmost, that they may acquit themselves with credit in the stations they fill: this exertion of the mind is, in the view I take of the subject, the efficient cause of the few generations an estate is enjoyed by such a family, or rather that such a family exists to possess it."

After this, we have some ingenious remarks on the gradual effects produced on the human figure and intellect, in proportion as bodily prowess or mental qualifications are more attended to by being more valuable to the possessor; but we cannot refrain from copying the conclusion of this chapter, as it shows the object of the author in a very striking point of view.

"There are many estates enjoyed by families that possessed them at the Norman conquest, but such families have never risen to greatness: they have not given birth to genius: it is in vain to enquire of them the improvements made even in agriculture, or of such as it is desirable should be made: they are men of precedents, not men of thought: the maxims of their ancestors guide them: they are useful in peopling a country, but not in civilizing it. Philosophers and poets, men of science or of taste, are not of this order; those who flourished a few generations back, live only in their works, their families are extinct. But it may be said, if this be the case, knowledge is a great evil, and the bulk of the people can never be informed. This remark will be attended to in another place; all I aim at, at present, is to establish the fact, that the most thoughtful people, taken as a body, are the least polific; by thoughtful, I mean any violent exercise of the mind, whether it be of a savage or a philosopher.

"Although this subject, from the nature of it, appears entirely confined to the human race, yet its principles are founded in the laws of nature, and may be supported by analogy. A hare, under the protection of a park, breeds earlier in the year, and yields more young in a season, than one that has been subject to perpetual alarm; the food of both may be abundant, but the timidity of the animal stifles the voice of nature. A mare, that ranges undisturbed over a barren pasture, brings forth its foal every season; but the same mare, well fed in a stable, and trained to the chace, is wholly unproductive. An elephant, caught at the earliest period of its life, and domesticated, and treated in a way apparently agreeable to it, never brings forth young. The same may be said of most species of birds; if they are allowed a large room to fly in, and live on the food they are most fond of, yet they never build a nest. These facts are sufficient to show, that an increase of animal life depends on something more than animal passion, or the abounding of the means of subsistence."

What we have offered, though in some degree explanatory of the object pursued in this truly valuable performance, will give but a small estimate of the various striking and original ideas to be met with in the work.

We should regret that the chapter on hereditary diseases is so short, did it not give us an opportunity of transcribing the whole.

"Hereditary diseases do not naturally and necessarily attend the human race. Leprosy, madness, gout, scrofula, sprung out of certain habits and practices; they were all acquired, and probably will be eradicated. Leprosy, originating in the want of personal cleanliness, has already given way to the improvements which have taken place in that respect: linen is now substituted for woollen in many articles of dress, and other regulations equally friendly to cleanliness, have caused leprosy almost to disappear. Madness is an increasing malady; it has its origin in anxiety, or it is the consequence of other diseases; it is sometimes wholly recovered from, but unfortunately a tainted constitution propagates its like, and no means have hitherto been discovered to prevent it. Celibacy is a virtue in persons whose progenitors have been thus afflicted: and even when it does not reach as far as madness, it may give a peculiarity of character and talent. I have sometimes enquired into the family history of youths of extreme dissipation, and so often have found some part of the family at one period or other afflicted with madness, that I now seldom pass my judgment of such characters till I am first assured they are moral agents. I have little doubt that madness is the cause of more vice than is imagined. The subject merits the attention of moralists. What parent can answer for the conduct of his child if this be the case? for education is but a feeble check to the slightest madness. Madness is an evil so various in its mode of operation, so difficult to remove, so distressing in all its branches, that it ought to be a subject of general care that it does not spread: as it had an origin, it may also have a remedy; if this be not discovered, the families that are afflicted must become extinct, for the world cannot be moralized so long as madness exists. The gout is not a disease of the poor, and need not be of the rich; it enters a family, or leaves it, at pleasure. The scrofula scrofula is the offspring of a cold and fickle climate: the plan adopted in many families from the advice of Locke, of attempting to make the children hardy by thin clothing and spare diet, has in many instances given birth to this malady; it makes its appearance at the close of winter, and is suspended by the warmth of summer. As soon as the malady is noticed, recourse is had to the liberal use of wine, and by this means the external appearance of the disease is occasionally removed; but may it not be doubted whether consumption has not sometimes had its origin in this practice? would it not be more advisable to clothe and lodge children warm, to keep them on nutritious diet, and at the age of 10 or 12 gradually to give up this plan, and substitute a diet sufficiently nutritious but less stimulant? By this means the disease is combated before it has disturbed the system; the constitution is strengthened, consumption is guarded against, and injurious habits are prevented. A scrofulous family is always consumptive. Should a young person have been drinking freely of wine to repel any external mark that may appear, and should symptoms of consumption take place, instantly wine and bark, and those remedies calculated to remove scrofula, are abandoned for milk and vegetables. Plans so opposite cannot be proper. That any regulations in diet will eradicate the disease I do not expect, but a better acquaintance with the properties and influence of the climate, as the cause of the malady, which the active spirit of research of the present day warrants the expectation of, may lead to other and more effectual remedies.

"I have dwelt thus long on this subject for the sake of pointing out these diseases as not being necessarily connected with the constitution of man.

"Contrary to what might have been expected, hereditary diseases are great promoters of population; the world is considerably more full in consequence of their existence. The Jews, through whose veins leprosy flowed, increased faster than any other nation is said to have done. In a few districts in the north of Europe, scrofula prevails as much as leprosy did among the Jews, and children are as numerous. Particular families, subject to either of these diseases, are commonly very prolific; it counteracts, in a considerable measure, the influence of those causes that have a tendency to occasion sterility, and keeps in existence names that would otherwise have been extinct.

"The gout may be acquired in any country, and becomes hereditary; but in cold climates it is most frequent and most severe. The Spaniards, afflicted with this disease, frequently remove to Mexico, as I have been informed, and find some mitigation of their suffering in the warmth of the climate. The Greeks were not unacquainted with the gout, and their opinion accorded with ours, that devotion to Bacchus at once gave birth to the malady, and disposed the martyr to a continuation of homage. Families that possess hereditary gout are seldom childless.

Madness

"Madness is a disease not altogether of the same nature as those just mentioned; it respects the connection of the mind with the body: and I have not sufficient knowledge of the subject to advance a confident opinion respecting its influence on fecundity.

"From the above facts and observations, it is evident that certain diseases so far influence the body as to be propagated, which could not happen if the principle of increase was, as Mr. Malthus intimates, a given quantity: that which is liable to alteration may be increased or diminished. Hereditary diseases, experience teaches, hasten those evolutions in the system that are necessary to pregnancy. If six children be as many as a marriage of sound and healthy persons usually yields, eight is not a larger proportion for those who are diseased. I would not be meant to imply, that all numerous families are infected with disease, that is not the case; but I do contend, that an unsound constitution, in a civilized country, most commonly proves prolific."

Nothing can be more just than most of the observations contained in this chapter: but we feel ourselves under the necessity of making a few remarks. We could have been very glad if our author, with his usual precision, had defined in what degree we are to consider diseases as hereditary. If by the term, "eradicate the disease," we are to consider it like a root, then all the branches must be expected to partake of the properties. But it is certain, that in many families the same causes which excite such diseases in some individuals, produce no such effect in others; the illustration therefore should be avoided. We would further remark, that in our opinion Dr. Jarrold presumes too much, or is not sufficiently explicit in conceiving, that, as madness had an origin, it may have a remedy, or that the world cannot be moralized as long as madness exists. If by the first expression is meant, that by avoiding every increase in families in whom madness has been found, we may rid mankind of this calamity, we can only remind him, that it has been felt by a personage in this country, in whose family, had it existed before, it would have been impossible that such an event should have been unknown. Nor is it quite certain that moral restraints are feeble even in something more than slight cases of madness. There is not an inhabitant of Bedlam who is not awed by the presence of a keeper, nor scarcely a madman in the kingdom who is not cowed at the appearance of a strait waistcoat. In this commercial country certain offices are assigned to each individual, and certain remunerations are expected. Mad people are doomed to hospitals, or to other receptacles, the keepers of which have not always an interest in the recovery of their patients. Were the tempers of each to be studied by those who could devote their time to so humane a task, we doubt not that the dangers of madness would be as rare in this country as in others, in which an attention to the infirmities of mankind is not so universally a mercantile pursuit. There is another most important consideration, which we recommend to the ingenuity of Dr. Jarrold. Are we bound bound by any laws, human or divine, to watch with so much assiduity the prevention of suicide in maniacs? Madness of itself rarely shortens human life, yet it is certain that in this, as well as other diseases usually called hereditary, nature has made a larger provision as if for a certain waste. As the work before us professedly reverts very often to ultimate causes, we may be allowed to submit to the the writer, whether as he seems to admit that the prolific constitutions of certain families may be necessary on account of the short life of many of its individuals, that the *race* in such a community should not become extinct; so may it not be gradually improved by the premature death of many, who would be the means of perpetuating the malady? A writer, whose work has lately come under our notice, remarks of the leprosy, that the subjects, though they continue to live, are rendered incapable of propagation by the common progress of the disease; and it is remarked by some of the best writers, that cancerous women are usually barren. Such facts imply an attention in nature to fix some limits to the propagation of incurable diseases, and probably the same might be traced in a less proportion in diseases, which, though curable, mark a constitutional disposition to fall into them from very slight causes.

These considerations we leave to the ingenuity of our author, whose work abounds with so much good sense and accurate reasoning, that we cannot help expressing our gratitude for the satisfaction we have found in its perusal.

---

*Observations on Medical Reform; illustrating the present Condition of Medical Science, Education, and Practice, throughout Great-Britain and Ireland; and proposing such Alterations therein, as appear most likely to succeed in Remedying the several Evils, which abound in this Profession, and which have, at length, become Subjects of universal Complaint*; 8vo. Dublin.

Medical Reform is so frequently talked of, and so little understood, that every attempt at explaining so complicated a subject deserves due attention. Before we enter on the present work, which takes up the question on a larger scale than most that has come before us, we shall offer a few remarks on a subject, concerning which, we may, hitherto, have appeared backward in expressing our sentiments.

Medicine has been very properly divided into the empirical and physical. The first is confined only to that experimental knowledge, by which a person having seen a malady cured by a certain remedy, conceives, that whenever such a malady occurs again, it will yield to the same remedy. The physician, on the contrary, is supposed to be so far conversant with the operations of Nature, as to ascertain the cause to which the remedy owed its success, and on any future occasion to judge of the propriety of using the same from his superior knowledge of such causes.

That

That both empirical and physical knowledge should be united cannot be questioned, nor, that to such an union we must look for the able and decided practitioner.

Another suggestion, which will arise in every reader's mind from the perusal of the work before us is, the too general impression, that the reverse of wrong must be right. In Great Britain, we are apt to complain of the union of pharmacy with any of the other branches. The author of these observations seems of the same opinion; but, whilst he wishes to confine the apothecary to the counter, he proposes, at the same time, that the surgeon and physician should be allowed to practice pharmacy, as far as extends to their own prescriptions. Now, let us ask, for a moment, what would be the consequence of such a law? The younger practitioners would dispense their own medicines, for which purpose they must have an assistant, of some description, at home; and are we certain, as their practice increased, that they would forego a profitable branch of their profession, which they could carry on with little trouble to themselves? Who then would remain to employ the apothecaries?

This opinion of our author seems to have originated in the manner in which the profession is divided in Ireland. The College of surgeons and physicians have interdicted their members from the practice of pharmacy, but the apothecary is unrestrained even from the important operations of surgery. No one can doubt the absurdity of this. But here, as in most other cases, it is much easier to object than to remedy.

It appears, by some prefatory remarks, that the OBSERVATIONS were first published in a Dublin Newspaper, and since collected in the form they are now offered to the public. They contain an account of the medical schools in the united kingdom, with many useful remarks; and others, which appear to us both hasty and crude. We shall not travel with the author through the whole, but content ourselves with a short extract from the concluding parts, and our remarks on the same.

"The legislature should by their decree acknowledge four departments as composing the medical profession, viz:—Physic, surgery, midwifry, and pharmacy.—To each of these a simple and efficient constitution should be given, of which I shall now attempt an epitome or outline.

"Physic should be committed exclusively to the Universities—of which a sufficient number should be constituted seminaries of medical education,—be empowered to examine into qualifications,—and to confer medical degrees.—Oxford and Cambridge universities for England, those of Edinburgh and Glasgow for Scotland, and the university of Dublin for Ireland, have been already shewn to be competent to this purpose.—These should, therefore, possess full and equal powers to execute this important trust; and no other medical schools should be acknowledged. Each university, so constituted, should possess the following professorships, as essential sential to the formation of a complete medical seminary—viz. a professorship of anatomy and surgery; a professorship of the institutes or theory of medicine, a course which comprises physiology, pathology, and therapeutics; a professorship of botany; a professorship of chemistry; a professorship of materia medica and pharmacy; and a professorship of the practice of physic. There should be an unappropriated professorship of clinical medicine. A professorship of natural history has been oftentimes suggested as necessary to a school of medicine; and certain acts of parliament, on the statute-book of this kingdom, actually establish it with this view. A professorship of midwifry, though hitherto, for reasons which are unexplained, utterly neglected in this kingdom, seems absolutely necessary."

In answer to this, we would ask, how is a practical art to be taught but by practice? and, however proper Edingburgh, Dublin, and even Glasgow, may be for the schools of the respective kingdoms in which they are situated; will any one say, that Oxford, or Cambridge should supersede London? Where can the "Practice of Physic" be so well taught, as where there are the greatest number of Hospitals? Where else, above all, can the Clinical Lectures be exhibited with any advantage?

As to the prevention of apothecaries from practising medicine, in our opinion the only error is, that they are not examined regarding their capacity for practising. Even the custom, as it now stands, is, in many respects, advantageous to the improvement of knowledge, though often injurious to individual patients. By the various practice which apothecaries have opportunities of seeing, they may often become the organs of information in consultation, and suggest hints, which the physicians may improve.

On the whole, though we are ready to admit, that this work contains much useful information, and is written in a very pleasing style, yet it comes, in our opinion, far short of that conviction, which the author, in some places, seems to demand.

